# We Need to Delay the Age of Onset of Alcohol Consumption

**DOI:** 10.3390/ijerph17082739

**Published:** 2020-04-16

**Authors:** Lucía Moure-Rodríguez, Francisco Caamano-Isorna

**Affiliations:** CIBER de Epidemiología y Salud Pública (CIBERESP), Department of Public Health, Universidade de Santiago de Compostela, 15782 Santiago de Compostela, Spain; lucia.moure.rodriguez@usc.es

**Keywords:** alcohol consumption, youth, alcohol-attributable fraction

## Abstract

In recent years, new consumption patterns, such as binge drinking, have increased among young people and have not always been recognized as problematic either by health personnel or by society in general, as they are intensive episodes, interspersed with no consumption periods. Although the prevalence of alcohol use disorders in the adult population continues to be higher in men than in women, these gender differences in relation to alcohol consumption are barely observed in adolescents between 14 and 18. Therefore, we are witnessing a change in the pattern of consumption, from regular to episodic, and an attenuation of gender differences. New patterns of alcohol consumption have not only been associated with an increased risk of alcohol use disorders in adult life, but also with neurocognitive involvement in youth. Understanding the risk and resilience factors of alcoholism or problematic drinking patterns will not only allow us to identify the most vulnerable group, but also to guide prevention programs towards protective factors; the skills that contribute to the natural abandonment of the pattern. Knowing the variables involved in the trajectories of abandonment and dependency would contribute to personalizing the interventions and increasing their efficacy and success—a lower relapse rate—, reducing the economic and socio-sanitary costs associated with alcohol dependency, as well as improving the health and well-being, family relations, work and social status of alcohol-dependent people.

## 1. Introduction

Alcohol consumption causes around three million deaths worldwide each year and can be attributed to more than 133 million disability-adjusted life years [1]. Alcohol use disorders imply a high physical and psychological burden not only on the affected people, but also on their families, work and social environment. A high proportion of multiple biopsychosocial problems are attributable to alcohol consumption: health problems like liver disease (48%), suicides (18%), interpersonal violence (18%), or intellectual disability (e.g., 100% of fetal alcohol spectrum disorders) [1]. The harms associated with alcohol consumption are especially severe in the WHO European region, where the percentage of disability adjusted life years attributable to alcohol is 10.1% in Europe vs. 5.3% globally [1].

In recent years, new consumption patterns, such as binge drinking, have increased among young people, but they have not always been recognized as problematic, neither by health specialists nor by society in general, as they are intensive episodes, interspersed with no consumption periods. Although the prevalence of alcohol use disorders in the adult population continues to be higher in men than in women, these gender differences in relation to alcohol consumption are barely observed in adolescents between 14 and 18 years old [2]. Moreover, higher prevalence is found among women for variables such as binge drinking or drunkenness. Thus, it seems that we are witnessing a change in the pattern of consumption, from regular to episodic, and an attenuation of gender differences.

Alcohol consumption during adolescence and early youth is framed in risk behaviors and the sensation-seeking characteristic of this evolutionary stage, associated with changes in neurodevelopment. From a neuropsychological perspective, the executive, salience, social cognition, and learning neural networks mature at different rates during this stage. Specifically, social cognition and control neural networks mature later than salience networks. Therefore, in the early phases of adolescents’ development, structural brain changes take place and lead to biased processing of affective stimuli (rewards, emotions, social information) and to decision-making based on short-term rewards, despite negative consequences for the future [3,4]. These characteristic behaviors of youth play an evolutionary role by facilitating the transition to adulthood, but they also present a risk factor for the development of alcohol use disorders when they start at an early age [5].

Paradoxically, despite the high prevalence of adolescents with a binge drinking pattern, most adolescents abandon binge drinking in early youth and only a few of them maintain an escalation towards alcohol abuse or dependence. The social plasticity hypothesis attempted to account for this paradox between risk and resilience of the adolescent brain [6]. Young people’s ability to learn from and adapt to the social environment relies on their development of socio-affective processing and brain plasticity during this stage, which consequently leads young people initially to a “social attunement” to the peer group, and then to the demanding adults. However, those who are involved in alcohol consumption, not to socialize but to decrease negative emotions (coping), may be less resilient and show maintenance or escalation trajectories towards alcohol use disorders instead of abandoning the consumption pattern.

A recent review highlighted the need to delve into emotional regulation and the moderating role of impulsivity in alcohol consumption [7]. Likewise, the evidence of differences in the pattern of neural activation between individuals with high risk of resilient alcoholism and those who are vulnerable concerning the mechanisms of emotional regulation has sparked interest in the identification of markers of resilience in alcohol addiction [8,9].

The relevance of attention bias in addictions, and specifically in alcohol dependence, is also well established [10]. Current research is aimed at systematically examining the role of attention bias in addiction and, especially, its predictive capacity of treatment outcomes (maintenance of abstinence). The role of attention bias for positive or negative words related to change has recently been analyzed, but the results require replication with greater depth of analysis, especially with respect to gender [11].

## 2. Alcohol Consumption in Youth and Consequences in Early Adulthood

During the last 15 years, our research group followed a cohort of university students with the objective of assessing the explanatory factors and the consequences of risky alcohol consumption and binge drinking. The analysis of the incidence rates of injuries in the Compostela Cohort allowed us to determine the effect of alcohol consumption on the university students, and today we can affirm that one in three injuries in young people could be avoided if we eliminate the practice of binge drinking [12]. Through the study of the association of risky sexual practices and alcohol consumption, we showed that 25% and 53% of these risky sexual practices are attributed to alcohol consumption in women and men, respectively [13]. Lastly, the monitoring of young people between the ages of 18 and 27 demonstrated that one in five traffic accidents is attributed to binge drinking [14].

The monitoring of the cohort of university students allowed us to characterize the evolution of risky alcohol consumption and binge drinking prevalence in people between 18 and 27 years old [15,16]. In this line, we found that risky consumption of alcohol and binge drinking decrease significantly at the age of 24, with higher prevalence rates and a later peak among men (22 years old) than women (20 years old). We also showed that the patterns of risky consumption and binge drinking between 18 and 22 years old could be pointing to a greater vulnerability to alcoholism in adulthood [16].

Furthermore, Macty et al. [17] analyzed the exposure to binge drinking at the age between 17 and 20 and alcoholism at the age of 30 in the American National Longitudinal Survey of Youth and found an increased risk in both men and women. In the United Kingdom, Viner et al. [18] also reported an association between exposure at 16 years old and alcoholism at 30. On the contrary, the Australian study published by Sillins et al. [19] found no association between binge drinking and alcohol dependence at age of 30. The inconsistencies presented by these studies are mainly because the confusion between frequency, quantity and consumption pattern is omnipresent, and even when adjusted for these variables, the presence of residual confusion is not always totally avoided.

Binge drinking patterns have not only been associated with an increased risk of alcohol use disorders in adult life, but also with neurocognitive involvement in youth. The recent systematic review of neuropsychological studies in young people reported that the binge drinking pattern is associated with cognitive difficulties, especially with lower performance in verbal memory and executive deficits [20]. The results provided by our group over the last decade show difficulties in performing tasks that involve inhibitory control, cognitive interference, working memory and episodic declarative verbal memory—functions dependent on frontolymbic and frontoparietal areas [3,20,21,22,23,24,25]. Finally, recent literature reaffirms the need for a systematic examination of the gender variable. Women with a stable binge drinking pattern for two years show a memory bias to emotionally negative words and lower performance due to the interference generated by the difficulties to disengage attention from those stimuli [26].

## 3. Manuscripts Selected by Us

**Figure 1:**
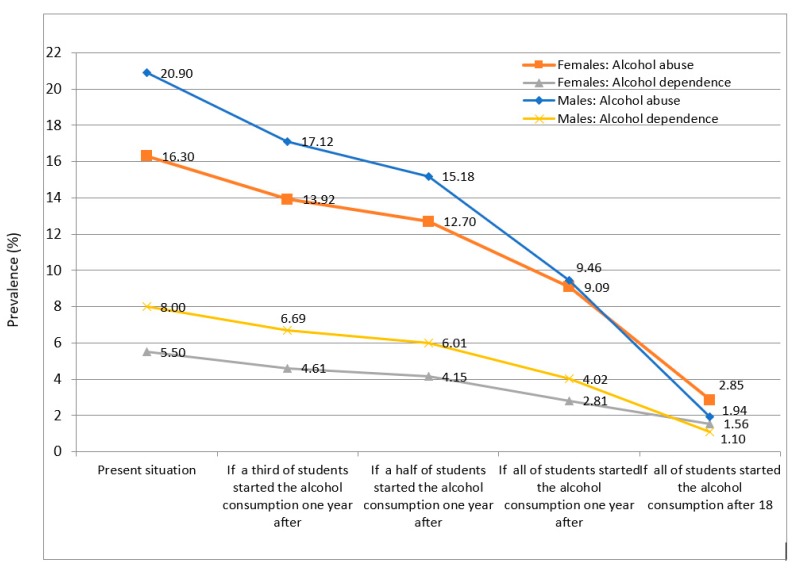
Estimated impact of the delay in the age of onset on alcohol abuse and dependence.

We have selected a total of nine manuscripts that delve into the alcohol consumption problem among young people and adolescents from different perspectives. Caamaño-Isorna et al. [27], using data from Spit for Science: The VCU Student Survey, examined the impact of the delay in the age of onset of alcohol on alcohol abuse and alcohol dependence, and estimated a reduction of around 75% of alcohol abuse and alcohol dependence if the consumption of alcohol started at 18 as established by law, as displayed in Figure 1. We need to delay the age of onset of alcohol consumption in our youth. This issue is one of the main factors—if not the main factor—in the prevention of alcohol use disorders.

The manuscript of Tabernero et al. [28] presents a study carried out between 50 groups of friends, in which the authors observed how the effect of drinking refusal self-efficacy predicts alcohol consumption behaviors, taking into account effects of peers´ enhancement motivation and protective behavioral strategies. In addition, considering environment and friends, Belzunegui-Eraso et al. [29] studied the influence of consumption by individuals with a close relationship to the consumer. Their results showed higher consumption rate among those whose families and especially whose friends consume alcohol. They also observed higher consumption among those who consider themselves better informed regarding substance use. 

The study by Herrero-Montes et al. [30] addressed polyconsumption among university students. Although the study did not find memory or executive function impairment, it reported a strong relationship with the studied substances. Following the polyconsumption line, Busto-Miramontes et al. [31] focused on assessing the relationship of alcohol consumption among university students with the nonmedical use of prescription drugs, and highlighted the importance of delaying the age of onset of alcohol consumption to prevent the polyconsumption.

Paramo et al. [32] analyzed the influence of polyconsumption on academic performance in a cohort of university students. The authors stressed on the importance of carrying out preventive measures focused on improving adaptation to academic life. In the same cohort, Blanco-Ramos et al. [33] studied how the motivational value of stimuli related to alcohol modulate the inhibition of the response in intensive consumers, through go/no go tasks.

Drabwell et al. [34] analyzed alcohol consumption after sudden bereavement by unnatural causes among adults aged 18 to 40. Their results show how men use alcohol to facilitate the expression of emotions, while in young people it was more common as a form of rebellion. Finally, Swahn et al. [35] showed interesting associations between alcohol and risky sexual practices in their study of adolescents from the Kampala slums. 

We hope that the selected manuscripts will help answer several of the main questions related to alcohol consumption in adolescents and young people. Understanding the risk and resilience factors of alcoholism or problematic drinking patterns will not only allow us to identify the most vulnerable people, but also to guide prevention programs towards protective factors and the skills that contribute to the natural abandonment of the pattern. Identifying the variables involved in the trajectories of abandonment–dependence can also contribute to personalizing the interventions and increase their efficacy and success—a lower relapse rate—, reducing the related economic and socio-sanitary costs, and improving the health and well-being, family relations, work and social status of people dependent on alcohol.
